# Reflections on mental health, *Ngozi*, and the *Dandemutande* approach in Zimbabwe

**DOI:** 10.4102/ajod.v14i0.1599

**Published:** 2025-06-09

**Authors:** Oliver Mutanga

**Affiliations:** 1 Graduate School of Education, Nazarbayev University, Astana, Kazakhstan; 2 Open Distance Learning Research Unit, College of Education, University of South Africa, Pretoria, South Africa

**Keywords:** *Ngozi*, mental health, Western perspectives, *Ubuntu*, *Unhu*, *Dandemutande* approach, Zimbabwe

## Abstract

**Background:**

Western mental health models prioritise biomedical explanations and interventions at the expense of indigenous non-Western belief systems that offer culturally relevant understandings of mental health. In Zimbabwe, *Ngozi* [reconciliatory and restorative spirits] play a significant role in shaping mental health experiences and perceptions. This article introduces the *Dandemutande* [Spiderweb] approach, an innovative framework that responds to the limitations of solely relying on the Western-based mental health conceptualisation by considering a multidimensional approach that acknowledges and respects cultural and spiritual dimensions alongside Western-based medical interventions to address mental health challenges in non-Western contexts.

**Objectives:**

This study aimed to explore the relationship between mental health challenges and *Ngozi*, and how *Ngozi* influences the understanding and management of mental health challenges in Zimbabwe.

**Method:**

A multi-layered, autoethnographic methodology integrating personal reflections, narrative accounts, the literature, and media analysis was employed to investigate how *Ngozi* is believed to affect mental health outcomes.

**Results:**

This article identifies *Ngozi* as a significant and influential factor within Zimbabwean cultures, which contributes to mental health issues.

**Conclusion:**

The findings introduce the *Dandemutande* approach to mental healthcare, which integrates cultural and spiritual dimensions with Western-based medical interventions to address mental health challenges.

**Contribution:**

This article highlights the importance of inclusive mental health practices that incorporate indigenous belief systems, for example, reparation and healing initiatives such as compensation. The proposed framework has the potential to positively contribute to mental healthcare in other non-Western contexts where mental health has cultural and spiritual dimensions.

## Mental health in Western and non-Western contexts

The global mental health environment is marked by significant disparities in understanding and treatment approaches (Taylor & Burgess 2019). This is primarily because of differences in how mental health challenges are conceptualised and the frameworks used to study them, among other factors.

Western mental health frameworks, largely shaped by biomedical models, tend to view mental health challenges through a primarily individualistic lens (Addo 2018; Chandler & Shera 1992). The Diagnostic and Statistical Manual of Mental Disorders (DSM), its variants, DSM-5 and DSM-5-TR, and the International Classification of Diseases (ICD) are widely utilised diagnostic tools that categorise mental challenges based on observable symptoms and individual behavioural patterns (Borgogna, Owen & Aita 2024; Utoblo 2017; World Health Organization 2024). These frameworks emphasise biological and psychological factors (Addo 2018; Brijnath & Antoniades 2018). Therapeutic approaches, such as cognitive behavioural therapy (CBT) and psychodynamic therapy, which typically focus on individual-level interventions aimed at modifying maladaptive thoughts, behaviours, or emotional patterns (Annelisa 2014) are predominantly employed. Emphasis is frequently placed on identifying and treating pathology with the goal of returning individuals to a perceived state of ‘normality’ as defined by Western societal standards (Taylor & Burgess 2019). Although this approach is relevant and effective in certain instances, it faces criticism for its inherent limitations when applied across diverse cultural contexts (Borgogna et al. 2024). For instance, the DSM-5 has been challenged for its potential inability to fully consider cultural differences in mental health diagnoses (Zagaria & Zennaro 2024). Moreover, the role of cultural narratives and language in shaping the definition and understanding of mental health issues is often neglected in these predominantly Western frameworks (Addo 2018). Furthermore, in Western traditions, there is a history separating the mind and the body, while perceiving mental health challenges (Stein et al. 2024). Currently, in these contexts, mental challenges are treated as medical issues – health conditions treatable only with medicines and therapies. This contrasts with numerous non-Western views that regard mental and physical health as intertwined aspects of holistic well-being (Meaden 2024). In addition, the focus on individual responsibility and self-reliance prevalent in Western societies can hinder help-seeking behaviour, particularly for individuals without adequate social support or resources to independently navigate the mental health system (Almu & Amzat 2024).

Most non-Western traditions’ understandings of mental health often emphasise the interconnectedness of the individual, family, community, and the spiritual realm (Almu & Amzat 2024; Kong et al. 2023; Molot 2017). For instance, many African cultures incorporate supernatural elements into their understanding of health and illness (Ayinde et al. 2023; Shange & Ross 2022). Mental distress is frequently linked to social, environmental, spiritual, or ancestral factors in addition to biological or psychological causes (Almu & Amzat 2024; Molot 2017), thus framing it as an issue that extends beyond the individual. Traditional healing practices, involving healers, spiritual leaders, and community members, play a central role in addressing mental health challenges (Berhe et al. 2024; Wüthrich-Grossenbacher et al. 2023). These practices often incorporate rituals, ceremonies, herbal remedies, and spiritual interventions aimed at restoring balance and harmony within the individual and their environment (Bartholomew 2016; Ngobe, Semenya & Sodi 2021).

The major difference between Western and non-Western conceptualisation lies in the aetiology and treatment of mental health challenges. Western models primarily focus on biological and psychological factors, employing interventions aimed at modifying individual-level processes. In contrast, traditional African approaches often consider social, environmental, spiritual, and ancestral influences, incorporating interventions that engage the entire community and spiritual realm (Kong et al. 2023). The views of mental illness in some African societies are deeply entrenched in socio-cultural contexts, including the attribution of mental illness to spiritual causes and reliance on traditional mental healthcare methods that engage the entire community and spiritual world (Almu & Amzat 2024; Kong et al. 2023). These divergent frameworks shape how mental health is understood and addressed globally, highlighting the need for comprehensive approaches (Taylor & Burgess 2019).

## Epistemic hegemony in mental health

The marginalisation of non-Western conceptualisations has resulted in a significant power imbalance, with Western models often dominating research, policy, and practice (Kelley 2021; Taylor & Burgess 2019). This is particularly true in the context of colonial psychiatry history, where Western psychiatric practices were often imposed on colonised populations, leading to a suppression of indigenous knowledge and practices (Jeater 2020a, 2020b). Colonial psychiatry sought to interpret mental health challenge symptoms in non-Western cultures, forming the basis for ethnopsychiatry while exposing the political agendas underlying the discipline (Buoli & Giannuli 2021). Different colonial empires employed varying approaches, with the British using psychiatry for political control and the French focusing on cultural assimilation (Buoli & Giannuli 2021).

The dominance of Western-centric mental health frameworks has had several significant consequences for non-Western societies. Firstly, it has led to a misdiagnosis and mistreatment of individuals whose experiences are not easily categorised within Western diagnostic frameworks (Ivbijaro 2010). Cultural behaviours and expressions of distress, when interpreted through a Western lens, may be misdiagnosed as pathological (Jarvis & Kirmayer 2021), resulting in mistreatment, poor outcomes, and limited access to culturally relevant care (Kelley 2021; Taylor & Burgess 2019). Secondly, it has undermined the value and validity of traditional healing and intervention practices, leading to their marginalisation and a loss of valuable indigenous knowledge (Kong et al. 2023; Taylor & Burgess 2019). This marginalisation is further compounded by limited mental health resources and infrastructure, particularly in the Global South (Almu & Amzat 2024). For example, the absence of culturally adapted, evidence-based interventions for Aboriginal youth in Canada demonstrates the gap between Western-centric approaches and indigenous perspectives (Williams & Mumtaz 2008).

Furthermore, Western models often prioritise individual-level interventions, overlooking the community and family support integral to mental well-being in non-Western cultures (Wright 2021). Similarly, Chandler and Shera (1992) highlight the differences in mental health conceptualisations between Pacific-Asian cultures and Western cultures. They suggest that Western-centric practices may overlook the familial and communal aspects that are integral to mental health in non-Western societies. This can lead to ineffective interventions that fail to address the underlying socio-cultural factors that contribute to mental distress (Williams & Mumtaz 2008). The emphasis on Western notions of ‘evidence’ can also lead to the marginalisation of Aboriginal knowledge and practices, impacting funding, programming, and opportunities for mental health and well-being in indigenous communities (Williams & Mumtaz 2008).

Western diagnostic categories and treatment protocols impose epistemic hegemony, privileging Western knowledge systems while silencing diverse perspectives (Noda 2020). This dominance is evident in cross-cultural research, where Western frameworks shape methodologies and interpretations (Kelley 2021), underscoring the need for culturally responsive practices in non-Western contexts.

Kong et al. (2023), emphasise the need for dialogue between Western and African mental health frameworks to foster understanding and respect for diverse healing practices. Kelley (2021) makes similar suggestion by calling for the incorporation of local cultural understandings and practices into mental health research to challenge colonial frameworks. In response to this call, this article seeks to answer the critical question: *What is the relationship between Ngozi and mental health challenges, and how does Ngozi shape the understanding and management of these challenges within the Zimbabwean context?*

### Ngozi

*Ngozi,* a Shona term for a spirit that is believed to inflict harm or illness as retribution for past wrongs (Jeater 2020a, 2020b), plays a significant role in the conceptualisation of mental health. Other scholars refer to *Ngozi* as avenging spirits, but just as Jeater (2020a) states, the reconciliation and reparation heal broken human relationships between individuals, and within families and communities. As such, I argue that the primary aim of these spirits is not to avenge but to restore relations; hence, I refer to them as reconciliatory and restorative spirits.

Samkange and Samkange (1980) note that:

Mashonas believe that when a person dies his spirit lives on. So, if a person is wronged and that wrong is not righted while he is still alive and the person dies while nursing a legitimate grievance, or is murdered, his aggrieved spirit will return, after his death, to avenge itself on the person or family of those who wronged him. Such an aggrieved spirit is called ngozi. Until propitiated, ngozi is unforgiving and uncompromising. (p. 51)

Unlike Western ideologies that differentiate between murder and accidental death, traditional Shona belief holds that the spilling of human blood, intentional or accidental, results in *Ngozi. Ngozi* seeks restorative justice, aiming to restore balance and fairness rather than acting out of spite or vengeance. This understanding reinforces the communal values and collective responsibility inherent in *Ubuntu* or *Unhu*.

As Gelfand (1973) explains:

There is the ngozi or angered spirit of a person murdered. Such a spirit will seek revenge on the members of the family responsible for the death whether it was intentional, unintentional or purely an accident. No one is permitted to kill. The well-being and right to survive is the prerogative of every human being. (p. 122)

Gelfand’s account highlights the understanding of *Ngozi* as a mechanism for restorative justice. This resonates strongly with historical and contemporary cases, such as those documented by the Catholic Commission for Justice and Peace (CCJP). Testimonies collected by the CCJP in Zimbabwe in the early 1980s referenced former soldiers tormented by *Ngozi*, who sought out the families of those they had killed to negotiate reparations and seek redress (CCJP 1997). In some instances, *Ngozi* cases reflect longstanding grievances that may date back decades or even centuries (Jeater 2020a). *Ngozi* is said to cause misfortunes, serious illnesses, or deaths within the perpetrator’s family until they acknowledge the wrongdoing and undertake the culturally recognised reparations for the affected family. This compensation often involves appeasing the *Ngozi* through reparations such as offering cattle to the victim’s family. The purpose is to act as a deterrent against wrongdoing, reinforcing social norms and the importance of communal responsibility.

The relationship between *Ngozi* and mental health is underexplored in literature. The specific characteristics and actions of *Ngozi* vary across different ethnic groups and traditional belief systems, but in most cases, mental distress or challenges are manifested. Some cultures view *Ngozi* as ancestors seeking justice for past grievances, for example, past killings, while others perceive them as independent entities acting on their own accord (Tobias et al. 2014). The actions of these spirits are often believed to be triggered by specific events or actions, such as transgressions against social norms, unaddressed familial conflicts, or unresolved disputes. These beliefs are not simply abstract concepts; they are integral to the daily lives of many individuals, influencing their experiences and perceptions of the world and their responses to adversity. Traditional rituals and ceremonies often play a crucial role in appeasing *Ngozi* spirits and restoring balance to the community (Tobias et al. 2014). These rituals might involve offerings, sacrifices, or cleansing ceremonies designed to mitigate the spirits’ anger and prevent further harm (Quiroz 2015). In Ghana, spiritual involvement in the treatment of illness and healthcare is deeply rooted in the culture (Asare & Danquah 2017). Mental conditions are often attributed to supernatural powers (Asare & Danquah 2017). This belief system, while potentially complicating diagnosis and treatment from a Western biomedical perspective, can also be a source of hope and facilitate recovery when integrated into treatment plans (Asare & Danquah 2017). Integrating *Ngozi* beliefs with modern mental health practices can significantly enhance treatment outcomes by addressing both spiritual and psychological needs. For instance, mental health interventions that incorporate traditional rituals alongside clinical therapies may foster greater community acceptance and individual engagement. This holistic approach not only respects culture but also utilises indigenous knowledge to create more effective and sustainable mental health solutions. By bridging the gap between *Ngozi*-based understandings and Western medical models, the *Dandemutande* framework, a culturally responsive mental health framework that views mental health support as an interconnected network (see the Discussion section for more information on the framework), exemplifies how culturally sensitive practices can lead to comprehensive care that honours both traditional and contemporary perspectives. This underscores the importance of culturally sensitive approaches that acknowledge and incorporate these beliefs.

However, while some researchers (Ashforth 2005; Niehaus 2013) dismiss *Ngozi* spirits as myths, such a position assumes that reality is only verifiable through certain empirical frameworks and overlooks other epistemological standpoints. For many communities in Africa, *Ngozi* is not a myth, but a lived reality that influences mental health and social dynamics (Mbiti 1969). In focusing solely on biomedical perspectives (Craddock & Owen 2010; Drysdale et al. 2017; Kapur, Phillips & Insel 2012), most studies run the risk of marginalising interpretations rooted in spiritual or cultural worldviews. Insel and Quirion (2005) contend that psychiatry’s public health impact requires understanding mental disorders as brain disorders, a stance reflected in the DSM-5’s symptom-based criteria (American Psychiatric Association 2013). While such biomedical approaches and clinical interventions remain highly relevant, they can be further enhanced when they respectfully integrate alternative epistemologies, such as the proposed *Dandemutande* approach, rather than disregarding them as mythical, thereby disrespecting and undervaluing them. Doing so moves beyond simplistic distinctions between ‘myth’ and ‘reality’ and acknowledges that spiritual beliefs, even if not subject to the same forms of empirical verification, are genuinely operative in people’s lives. This is not to reject scientific inquiry, but rather to argue that a multiplicity of knowledge systems should be recognised and valued. Acknowledging that *Ngozi* is integral to certain cultures, affirms that these beliefs deserve attention precisely because they shape experiences of health, well-being and community.

In contrast, scholars such as Whyte and Ingstad (1995) and Kirmayer (2006) argue for the inclusion of cultural contexts to better understand mental health and disability experiences. Kirmayer (2006) emphasises that mental health cannot be fully comprehended without considering the cultural environments in which individuals live. In disability studies, Goodley (2014) highlights the contributions of non-Western scholars, arguing that different colonial histories and the suffering of indigenous groups lead to diverse understandings of disablism. Such an epistemological perspective is essential for recognising the influence of *Ngozi* on how mental health and disability are understood and experienced across different communities. By engaging with and incorporating indigenous knowledge systems, mental health professionals can offer interventions that are culturally sensitive and more effective (Patel & Prince 2010; Whyte & Ingstad 1995). This understanding disrupts the dominance of Western biomedical paradigms and encourages a pluralistic view of mental health that honours the diverse experiences and beliefs of various populations.

Building on these scholarly insights, historical and contemporary cases further illustrate the profound impact of *Ngozi* beliefs on mental health and community dynamics in Zimbabwe.

## Zimbabwe: Mental health challenges and *Ubuntu*

In Zimbabwe, especially among the Shona tribal groups, *Ngozi* play a significant role in mental health and disability (Mugumbate & Mtetwa 2014; Mukushi, Makhubele & Mabvurira 2019). In contemporary Zimbabwe, mental health challenges are prevalent. Research indicates substantial prevalence rates of mental disorders, including depression and anxiety (Chibanda et al. 2016; Langhaug et al. 2010). The estimated treatment gap for major depression is 67% (Kohn et al. 2004). Zimbabwe counts a mere 16 psychiatrists (Chibanda et al. 2015), and traditional healers remain the initial point of contact for nearly one-third of patients (Patel, Simunyu & Gwanzura 1997). As in many other African contexts, the understanding of mental health in Zimbabwe is profoundly informed by cultural and spiritual beliefs (Potgieter & Van Rooyen 2018). For instance, mental distress might be attributed to ancestral spirits, witchcraft, or imbalances in spiritual energy (Potgieter & Van Rooyen 2018). The preference for traditional healing methods is often high in these contexts, with individuals frequently seeking help from traditional healers before engaging with, or alongside Western medical professionals (Molot 2017; Potgieter & Van Rooyen 2018). The use of faith-based and traditional healing methods for mental disorders in African contexts is well-researched, but normative responses often fall into two camps: those focused on the biomedical model and those adopting a more local perspective (Kong et al. 2023). This dichotomy between universalist and relativist approaches reflects the tension between Western and non-Western mental health frameworks.

Traditional African societies, including those in Zimbabwe, perceive reality as a dual construct comprising both material and spiritual realms. Human beings embody this duality, existing simultaneously as matter and spirit. Life is viewed as an enduring continuum that transforms from one form to another; thus, death is not an end in itself but a significant transition from physical existence to a purely spiritual reality. This perspective explains the numerous rituals associated with death among Zimbabwean ethnic groups, all aimed at assisting the enduring spirit in entering a higher spiritual realm.

Within this spiritual framework, ancestral spirits hold considerable power over the material world and are believed to have control on the living. This belief is central to understanding the concept of *Ngozi* among the Shona people of Zimbabwe. *Ubuntu* or *Unhu* philosophy, emphasising communal relations and harmony, provides the guiding principles for human interactions and underpins this understanding. Justice and fairness are cornerstones of these communities, and social equilibrium is maintained through cooperative effort and mutual assistance. When this balance is disrupted, the community is expected to take restorative actions to repair relationships and restore harmony, as failing to do so can lead to disorder and conflict.

Ndabaningi Sithole (1972:36–37), in his novel, *The Polygamist*, shows *Ubuntu*-based justice in action within the Zimbabwean traditional contexts. In an extremely unusual case in which Matutu’s son committed the grave act of sleeping with one of his father’s wives, a violation that, according to Ndebele tradition, was equivalent to sleeping with one of his mothers. The community saw this not just as a private transgression, but as a public threat to societal norms and stability. The presiding judge, Mlotshwa, explained the gravity of the offence (Sithole 1972):

This one is a crime against the people. It breaks the custom of the people. It threatens every home. If this evil is allowed to go on unpunished … we shall have poisoned ourselves. (p. 36)

Declaring the son as having ‘become a wild animal’, Mlotshwa ordered him tied to a tree and flogged with reins, tools normally reserved for taming cattle. This act was not merely punitive but symbolic, aiming to rid the young man of his ‘wild animal’ tendencies and reintegrate him into the community. After the punishment, Matutu’s son tearfully repented and promised to reform.

As illustrated by this case, *Ubuntu* or *Unhu* places the group above the individual, asserting that the community imparts humanity to its members (see Gade 2011; Metz 2023; Mutanga & Haihambo 2024; Samkange & Samkange 1980). This is encapsulated in the saying ‘I am because we are; We are because I am’, which carries profound implications for social responsibility. If a family member commits an offence, such as Matutu’s son, or a grave act like murder, the entire family or community bears collective responsibility. This collective responsibility reflects the belief that an individual’s behaviour can bring misfortune or calamity upon their entire lineage. Thus, community members are expected to uphold moral conduct, and an individual’s private life becomes the concern of the wider family – blurring the private-public life distinction (Mutanga 2025). Against this backdrop, the concept of *Ngozi* emerges as a critical mechanism for maintaining justice and social equilibrium. When someone is killed, the victim’s family suffers the loss of their lineage’s potential growth and continuity. In the Shona novel *Muchadura* [You Shall Confess], Emmanuel Ribeiro (1967) illustrates how the *Ngozi* of a murdered person can bring unaccountable misfortunes upon the murderer and their family, serving as both punishment and a demand for justice. The novel focuses on the murder of Chipo who was murdered by Tavengwa, her husband. Chipo returns as *Ngozi* and uses her daughter, Muchadura, as a medium to seek justice. Chipo’s *Ngozi* causes a series of tragedies. Overwhelmed by the events, the Tavengwa’s family ultimately confesses and pays recompense for the murder. This narrative reflects the Shona people’s belief in the sanctity of life, which underpinned *Ngozi’*s role as a traditional justice system. However, these indigenous practices were disrupted by colonial intervention. In 1899, the colonial government in Rhodesia enacted the *Witchcraft Suppression Act,* aiming to suppress African cultures, belief systems, and unwritten moral and legal frameworks (Vambe 2009). By applying constitutional law exclusively to white people while subjecting black people to both customary and constitutional law, the colonial government marginalised systems such as *Ngozi*, weakening their authority and eroding social cohesion.

This exploration of *Ngozi* and its role in maintaining justice and social cohesion highlights the profound impact of indigenous practices on community life. To further examine these dynamics, the following section outlines the methodology employed in this study, detailing the approaches used.

## Methodology

This study employed a multi-layered methodology, combining personal reflections, narrative accounts, literature, and media analysis to examine how *Ngozi* beliefs shape mental health perceptions in Zimbabwean communities. The methodological approach was designed to capture personal, cultural, and societal dimensions of these beliefs and their impact on mental health. Some key stories emerged during an ethnographic study I conducted in northern Zimbabwe, offering valuable insights into how *Ngozi* beliefs influence perceptions of mental health. Although this was not the original focus of the study, this account became a foundational aspect of this article. Drawing on my lived experiences as a Shona individual raised in Zimbabwean villages, I used personal reflections to contextualise *Ngozi* beliefs and their role in everyday life. These reflections provided a lens through which to interpret broader community perspectives. To complement these reflections, I conducted a detailed review of literature, including Zimbabwean novels and ethnographies, which offered cultural narratives and historical perspectives on *Ngozi* and its relationship to mental health. In addition, newspaper articles and media stories were analysed to understand how *Ngozi* is represented in public discourse and how its role in mental health challenges is discussed in Zimbabwean society.

The data collected from narratives, personal reflections, literature, and media analysis were analysed thematically, guided by Braun and Clarke’s (2006) framework which involved familiarisation with the data, generating initial codes, searching for themes, reviewing themes, defining and naming themes, and writing up findings. This approach allowed for a systematic and thorough examination of the data, ensuring that emerging themes were grounded in the content. One central theme was the role of *Ngozi* in enforcing communal responsibility. Stories and reflections highlighted how *Ngozi* beliefs embed accountability within families and communities. For example, during fieldwork, a narrative was shared about a man whose mental health challenges were attributed to *Ngozi* stemming from an unresolved murder in his family. Community members explained that the *Ngozi* did not seek reparations from the individual experiencing mental challenges, but from the whole family, reinforcing the *Ubuntu* or *Unhu* philosophy of shared responsibility. This theme demonstrates how *Ngozi* beliefs extend beyond individual behaviour, positioning the family and community as collectively responsible for addressing wrongs and maintaining balance. The fear of *Ngozi* serves as a deterrent, encouraging ethical behaviour to prevent spiritual disturbances and associated challenges, including mental health.

Another theme was how *Ngozi* beliefs shape interpretations of illness, mental health, disability, and death. Narratives and media accounts revealed that mental distress is often attributed to spiritual causes, such as ancestral grievances. For instance, one story recounted how a man’s mental health challenges were linked to *Ngozi*, believed to have been triggered by his family’s failure to comply with instructions from the spirit of a deceased former headman.

The final theme explored the connection between justice, reconciliation, and mental health. Rituals associated with *Ngozi* were seen as not only addressing past wrongs but also fostering healing for both the afflicted individual and the wider community. For example, a common belief is that if a murder remains unaddressed, the spirit of the victim will continue to wreak havoc until justice is achieved, often through reparations or public acknowledgement of wrongdoing. This theme highlights the dual function of *Ngozi* rituals: they provide justice for the wronged and reconciliation for the wrongdoer and their community. By resolving spiritual grievances, these rituals can alleviate mental distress attributed to *Ngozi* and restore harmony within families and communities. This process underscores the importance of integrating culturally relevant justice mechanisms into mental health interventions, particularly in contexts where spiritual interpretations of illness are prevalent.

The synthesis of personal reflections, narrative accounts, literature, and media analysis, combined with thematic analysis, provides a holistic understanding of *Ngozi* beliefs and their relationship to mental health. These findings inform the development of the *Dandemutande* framework, a culturally responsive model that integrates the spiritual and communal dimensions of *Ngozi* into mental health interventions.

The following section presents the findings of this study, offering a deeper exploration of the identified themes and their significance in understanding how *Ngozi* beliefs shape mental health experiences and responses in Zimbabwean society.

### Ethical considerations

Ethical approval to undertake the ethnographic study reported in some sections in this article was granted by the Zimbabwe’s Mashonaland Central provincial government and the local traditional leaders. Ethical clearance number ADM 27/18 was received on 21 June 2022.

## Results

This section presents the findings of the study, focusing on the interplay between *Ngozi* beliefs, mental health, and community dynamics. Through personal reflections, ethnographic accounts, and documented cases, I explore how *Ngozi* manifests in individual and communal experiences and how these beliefs inform local understandings of justice, responsibility, and well-being.

### Personal reflections on Ngozi

Growing up in a Christian household, I was taught that the dead know nothing, a belief rooted in biblical teachings. Yet, paradoxically, the same scriptures acknowledge the existence of spirits, often labelling them as evil. This duality in my religious upbringing left me both sceptical and intrigued by stories of the supernatural, especially those involving *Ngozi*. Most people in our village were Christians, so *Ngozi* was referenced as evil spirits, and the view was that they could not defeat Christ’s power. So, although we heard rumours of families suspected to have *Ngozi* (in one case, the entire family perished, except for the mother and her surviving grandchildren; this is unusual even for me), I do not remember hearing about traditional healers visiting them. At least, if they did visit them, they did so under the cover of the night.

To further illustrate how *Ngozi* beliefs shape understandings of mental health within communities, I now present specific cases from Zimbabwe. These stories underscore how *Ngozi* beliefs deeply influence perceptions, responses, and social dynamics related to mental health challenges.

### Stories of Ngozi and mental health

The narratives presented here are drawn from various sources to illustrate how *Ngozi* beliefs influence perceptions of mental health. The Chapoto story is derived from my ethnographic fieldwork in northern Zimbabwe, capturing first-hand experiences and community interpretations, while the Chokuda and Maziofa cases are based on media reports, reflecting broader public discourses on *Ngozi* and mental health.

### The man at Chapoto

One afternoon, during my ethnographic studies in northern Zimbabwe, I encountered a man whose untidy appearance and erratic behaviour suggested he was grappling with mental health challenges. Locals shared his story, explaining how his condition deteriorated after visiting his mentally ill brother, whose own symptoms subsequently improved. This exchange of affliction puzzled the community, who attributed the events to *Ngozi*. Now, his family fears visiting him, worried that the affliction might transfer back or spread further. They rely on the kindness of community members to check on him and provide food. Remarkably, he often walks the treacherous 65-kilometre stretch between Chapoto and Angwa business centres, an area teeming with wild animals such as elephants, buffaloes, and lions. Yet, he remains unharmed. It is as if an unseen force protects him. The family’s reluctance to visit him, fearing the affliction might transfer back, reflects how *Ngozi* beliefs shape familial and communal responses to mental health challenges. The story illustrates how *Ngozi* beliefs embed a sense of shared responsibility and fear within communities, influencing how they interpret and manage mental health challenges.

### The Mozambican herdsman

As we went deeper into discussions about mental health and *Ngozi*, another story emerged. It was about a refugee from Mozambique who had worked as a herdsman for a family in another part of Zimbabwe. During the Mozambican civil war in the 1980s and early 1990s, many Mozambicans fled to Zimbabwe, some finding refuge in camps such as Nyangombe near my village, others integrating into local communities, offering their labour in exchange for money and food. This particular herdsman made an agreement that he would work in exchange for cattle. After he had toiled for years, and as his herd of cattle grew, he expressed a desire to return to Mozambique with his livestock. The family he worked for initially agreed, but later, driven by greed, plotted to reclaim the cattle. They pursued the herdsman and murdered him along the way. What followed was a series of misfortunes that befell the murderer’s family. The avenging spirit of the slain herdsman began to torment their children, manifesting through unexplained illnesses and disturbing occurrences. The *Ngozi* spirit possessed a family member and demanded justice. The spirit’s terms were explicit: it wanted a wife as compensation. The family was required to take their own daughter to the herdsman’s relatives in Mozambique. The spirit gave them the directions. Desperate and terrified, the mother chose to sacrifice her stepdaughter instead, taking her across the border to fulfil the demand. Tragically, upon arrival in Mozambique, on their way, the mother began exhibiting severe mental health challenges. To this day, her whereabouts remain unknown. This incident underscores the impact of unresolved spiritual grievances on individuals’ well-being. Furthermore, it highlights the role of *Ngozi* as a mechanism for enforcing justice and reconciliation, with direct implications for mental health in the affected community.

Despite my Christian upbringing, where the African traditional supernatural is often dismissed or demonised, I could not help but acknowledge the patterns emerging from such narratives. I had come across numerous accounts on YouTube^1^ and in newspapers where *Ngozi* was manifested, including before traditional chiefs. One such case is the Chokuda case.

### The Chokuda case

In 2008, Moses, an opposition party member, was murdered by Foster and Farai Machaya, the sons of the Midlands Provincial Governor, Jason Machaya; Abel Maphosa, and two brothers, Edmore and Bothwell Gana – individuals connected to a ruling party official (NewsDay 2012). Following his death, his *Ngozi* began haunting the perpetrators and their families, demanding justice. Extraordinary events occurred. The first strange occurrence involved Governor Machaya’s daughter. She inexplicably left home and sought refuge in a graveyard. When confronted, she claimed she was with the late Moses. The second incident saw the local police attempting to bury Moses without his father’s consent. However, they were met with an inexplicable force: 10 officers tried to lift the coffin, but it would not budge. The third incident was the sudden mental breakdown of the magistrate who granted bail to the accused. Turmoil persisted until traditional reparations were made, including the payment of 35 cattle and US$15 000 by the Machaya’s family to Moses’s family. Only after this act of restitution was Moses laid to rest, and peace restored (The Newsday 2020).

This case reveals how *Ngozi* beliefs continue to hold communities accountable, offering a cultural framework for understanding justice, reconciliation, and mental health.

### The Maziofa case

In 2021, the Maziofa family from Rusape confessed to the 2014 murder of school teacher Bernard Chigwese after years of torment by his *Ngozi*. Following the crime, the family concealed the murder, but they began to experience a series of misfortunes, including unexplained illnesses, deaths, and mental health challenges (The Eye 2021). In one instance, a boy reportedly hallucinated, crying out, ‘Makandiurayirei?’ [*Why did you kill me?*] before tragically taking his own life. Overwhelmed by these events, the family sought help from traditional leaders, admitting their guilt and requesting mediation with Chigwese’s family. As one family representative explained:

They [*the perpetrators*] were arrested, but we ensured that the case died a natural death. However, Chigwese’s spirit is now haunting our family, we no longer know any peace. The family is now experiencing misfortunes all the time, while some are developing mental illnesses. (Kadzura 2021)

These findings reveal the profound influence of *Ngozi* on individual and communal mental health, justice, and social harmony. They challenge conventional Western approaches to mental health by highlighting the importance of culturally embedded beliefs. These insights inform the development of the *Dandemutande* framework, which seeks to harmonise indigenous beliefs with contemporary mental health practices to foster holistic well-being. In the following section, I explore how these insights can inform the development of holistic mental health interventions that bridge traditional practices and contemporary approaches.

## Discussion

Reflecting on these experiences, I recognise the importance of integrating spiritual or cultural understanding into discussions about mental health. While the reported narratives underscore *Ngozi* as a deterrent against wrongdoing and highlight the collective responsibility integral to *Ubuntu* philosophy (Mbiti 1969), it is not intended to suggest that all mental distress stems from personal or familial misdeeds. In many African cosmologies, what appears as ‘blame’ is often an acknowledgment that individuals are embedded within broader community networks, and that wrongdoing, even by extended family, may have communal consequences (Samkange & Samkange 1980). However, it is crucial to stress that *Ngozi* can be interpreted differently by different people. By recognising this, we can avoid conflating specific moral or spiritual interpretations with a universal ‘blaming’ of mental health challenges or disabilities, while still appreciating how cultural beliefs about responsibility and interconnectedness inform understandings of mental health.

In retelling these stories, my aim is not to verify or dismiss the supernatural, or to blame individuals or families, but to portray how spiritual beliefs shape understandings of justice and responsibility. For communities that uphold these beliefs, the occurrences are both real and meaningful. These cases exemplify the profound impact that *Ngozi* can have on both individual mental health and community dynamics, highlighting the necessity for integrated mental health interventions that respect and incorporate indigenous spiritual frameworks. However, despite the rich historical narratives and literary explorations of *Ngozi*, there remains a notable absence of research examining its contemporary impact on mental health in Zimbabwe. Most studies to date are historical in nature or presented through novels, which, while valuable, do not provide current practices and perceptions. Recognising this gap, this study seeks to investigate how beliefs in *Ngozi* influence the understanding and management of mental health challenges within Zimbabwean communities. Integrating *Ngozi* into mental health interventions is crucial for effective care. The Friendship Bench, a mental health intervention developed in Zimbabwe in 2006, offers a promising model for community-based care. This programme, influenced by the indigenous practices of family aunts who acted as advisers and counsellors, provides problem-solving therapy through trained lay health workers known as ‘Grandmothers’, who offer culturally adapted support (The Friendship Bench 2024). The Friendship Bench programme, influenced by indigenous practices, demonstrates how integrating traditional advisory roles with clinical therapies can enhance community engagement and treatment efficacy. This model serves as a precursor to the *Dandemutande* framework, which seeks to further harmonise traditional beliefs with modern mental health practices.

The profound influence of *Ngozi* on social harmony and individual well-being in Zimbabwean communities highlights a critical intersection between cultural beliefs and mental health. Despite the significant role that *Ngozi* plays in shaping perceptions and responses to mental health challenges, there is a noticeable gap in the integration of these indigenous beliefs into formal mental health interventions. This disconnect not only hampers the effectiveness of mental health services but also overlooks the rich cultural frameworks that can contribute to more holistic mental healthcare.

These stories highlight how deeply our cultural beliefs, spirituality, and mental health are interconnected in Zimbabwe. It is important to clarify that within many African cosmologies, mental distress can sometimes be attributed to past wrongs (Mbiti 1969), not necessarily those of the individual directly affected, but possibly of extended family or ancestors. While sharing these accounts does not imply that all mental challenges result from wrongdoing, my experiences in these communities suggest that this perspective is genuinely held and used to explain certain forms of distress. At the same time, I acknowledge that attributing mental illness to spiritual causes may risk stigmatising the individual. My goal is neither to impose this worldview nor to dismiss other explanations, but to convey how beliefs about communal interconnectedness inform local understandings of mental health. This will ensure that cultural beliefs are acknowledged and integrated as appropriate, facilitating engagement in treatment while respecting the values of the individual and community.

The experiences of individuals such as the man at Chapoto business centre demonstrate that *Ngozi* plays a significant role in our society, influencing how we understand mental health, illness, justice, and our relationships with each other. These accounts challenge the conventional Western approaches to mental health interventions I have encountered, which often emphasise clinical diagnoses and treatments while overlooking the cultural and spiritual dimensions integral to our context. Dismissing these beliefs not only alienates those who hold them but also potentially hinders the healing process. I find myself caught between two worlds: the Christian teachings of my youth and the undeniable presence of cultural beliefs in *Ngozi*. Whether labelled as a myth, evil or not, the existence of *Ngozi* is a reality in many communities in Zimbabwe, underscoring the need for mental health approaches that fully address our cultural context.

**FIGURE 1:**
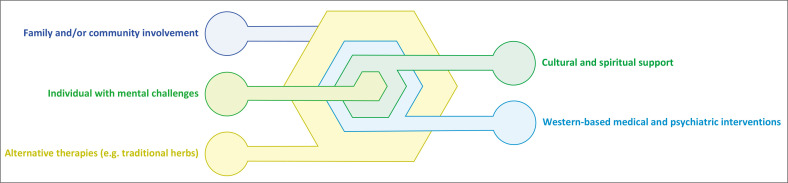
*Dandemutande* [Spiderweb] framework.

The stories of the man at Chapoto business centre, the tormented family haunted by the herdsman’s *Ngozi*, the Maziofa and the Chokuda cases, are not just tales of the supernatural; they are narratives that highlight the intersection of spirituality, justice, and mental well-being. They call for a more comprehensive approach that respects and incorporates *Ngozi*, rather than dismissing it outright. The Chokuda case exemplifies the need for interventions that address both spiritual and psychological dimensions, a core principle of the *Dandemutande* framework (Figure 1). By integrating legal accountability with traditional rituals, the framework ensures holistic justice and mental well-being.

The *Dandemutande* framework emerged from my analysis of the data that highlights the spiritual or cultural dimensions of mental health. It offers such an approach, drawing its conceptual inspiration from the intricate and interconnected nature of a spiderweb, known in Shona as *Dandemutande*. The individual experiencing mental health challenges is positioned at its centre. Surrounding the individual are various strands representing interventions from both Western and African traditions, including spiritual support, medical and psychiatric interventions, community and family involvement, and alternative therapies.

The *Dandemutande* framework (Figure 1) visualises mental health support as a multidimensional network where each strand represents different interventions and support systems. The intervention strands of the spiderweb are not fixed but dynamic, allowing for interventions to alternate and adapt based on the individual’s mental condition and underlying causes. This flexibility acknowledges that mental health is not static and that support mechanisms must be responsive to change. At the heart of the *Dandemutande* lies the person with mental health challenges, emphasising the individual’s significance and the personalised nature of care required.

Spiritual support incorporates indigenous beliefs, rituals, and practices such as acknowledging the role of *Ngozi* and engaging traditional healers or spiritual leaders. Medical and psychiatric interventions utilise Western medical practices, including psychotherapy, psychiatric evaluations, and medication when necessary. Alternative therapies incorporating herbal remedies that align with the individual’s beliefs and preferences are also important.

The *Dandemutande* framework’s strength lies in its holistic and integrative nature, broadening the available interventions and ensuring that care is comprehensive. By recognising multiple forms of healing and support, the framework expands the options available to individuals. Integrating traditional beliefs with modern interventions respects the individual’s cultural heritage, which can enhance engagement and effectiveness. For instance, acknowledging the role of *Ngozi* can address underlying spiritual concerns that might be overlooked in Western models. *Ngozi*, as a mechanism for communal responsibility informs the framework’s emphasis on community and family involvement, ensuring that mental health interventions engage the broader social network surrounding the individual. Notwithstanding the value of Western-based interventions, the man at Chapoto business centre appears to need spiritual intervention as a matter of priority. Moreover, the framework encourages different healing practices to work in concert rather than in opposition. This collaboration can lead to more effective outcomes as it addresses the physical, emotional, spiritual, and social dimensions of mental health. The central focus on the individual allows for personalised treatment plans tailored to the unique needs, beliefs, and circumstances of each person.

The *Dandemutande* framework bridges Western medical interventions and African traditional practices by recognising the interconnectedness of mental health, spiritual reconciliation, and communal well-being. This holistic framework addresses the limitations of isolated approaches by weaving together diverse healing practices to achieve meaningful, culturally resonant solutions. While some may argue that integrating traditional beliefs could complicate clinical practices, the *Dandemutande* framework addresses this by providing structured guidelines that harmonise both approaches, ensuring comprehensive and respectful care.

**FIGURE 2:**
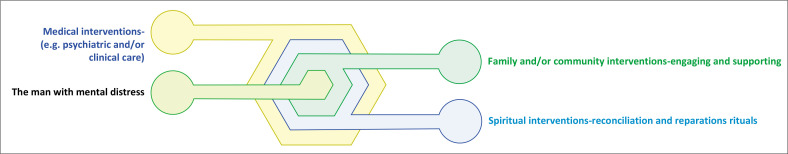
*Dandemutande* framework-informed mental health intervention approach.

In the case of the man who was allegedly having mental health challenges I saw at Chapoto business centre, the family might first consult with traditional healers to determine the demands of the *Ngozi* and take necessary steps to address them, such as offering reparations or performing rituals for reconciliation. Alongside this spiritual intervention, family members experiencing mental health challenges or illnesses can seek treatment at healthcare centres for psychiatric or medical care as shown in Figure 2.

The *Dandemutande* framework would have resolved the Chokuda case by running the legal and spiritual processes concurrently. While court prosecutions ensure accountability for the perpetrators, traditional rituals would address the spiritual dimension to appease the *Ngozi* spirit. This dual pathway prevents the situation from escalating into further spiritual harm, such as the mental distress experienced by the governor’s daughter. Both families would also engage in counselling and traditional healing rituals to foster reconciliation and emotional healing. This collective, integrated process strengthens the community fabric and ensures holistic justice for all involved.

For families experiencing recurrent mental distress, illnesses, or unexplained deaths, even in the absence of manifest *Ngozi*, the *Dandemutande* approach encourages proactive consultation with traditional healers to rule out unresolved ancestral grievances. At the same time, mental health centres can integrate culturally sensitive screening questions to identify whether *Ngozi*-related issues may be a contributing factor. By addressing both spiritual and biomedical dimensions early, families can avoid compounding distress and achieve more comprehensive care.

Community and family involvement is essential in utilising the *Ubuntu* or *Unhu* philosophy to engage members in the healing process. This approach reinforces social support networks that are vital for recovery, aligning with *Ubuntu* or *Unhu’s* emphasis on communal relations and harmony. The ‘we’ invoked in *Ubuntu* incorporates individuals, families, society, the living, the non-living, and non-human entities (Mutanga & Haihambo 2023), all of whom are important in mental health interventions. Some critics argue that *Ubuntu* philosophy suppresses individual freedoms (Gyekye 1997; Matolino & Kwindingwi 2013). However, this perspective overlooks the balance *Ubuntu* seeks between individual and collective well-being. Stories in this article reflect why the Shona people take interest in everyone’s affairs, as personal actions can influence the harmony of the whole family and society.

Implementing the *Dandemutande* framework has significant implications for mental health practice. Policymakers can design inclusive policies that incorporate indigenous practices and promote integrated mental health models. Mental health professionals can be trained to understand and incorporate cultural beliefs into their practice, enhancing their cultural competence. The Friendship Bench concept discussed earlier, which took the role of traditional aunts, is a powerful example of how traditional approaches can be incorporated into contemporary systems. Elsewhere, Mutanga and Haihambo (2024) have discussed how Rwanda successfully used its traditional community courts to fast-track the genocide trials. Empowering communities to take an active role in mental health interventions fosters ownership and sustainability of mental health initiatives. If such successful integrations are possible, then interventions informed by beliefs around *Ngozi* can likewise be woven into mental health initiatives. Recognising and embracing these indigenous interpretations do not negate the importance of Western methods; rather, they complement them by engaging local spiritual and cultural understandings of mental distress. Empowering communities to take an active role, whether through spiritual frameworks like *Ngozi* or culturally adapted counselling models, can increase the ownership, relevance, and long-term impact of mental health interventions.

By integrating culturally adapted interventions such as the Friendship Bench alongside spiritual dimensions, the *Dandemutande* Framework demonstrates the potential for diverse indigenous perspectives to coexist with Western mental health practices. Policymakers can use it to design inclusive policies that acknowledge indigenous knowledge alongside biomedical perspectives, thereby promoting integrated mental health models. Mental health professionals can be trained to understand and incorporate these cultural beliefs into their practice, enhancing their cultural competence.

An essential aspect of this study is its grounding in decoloniality theory, as articulated by Tuck and Yang (2012). Their perspectives were crucial in guiding research processes that not only explore but also respect and advocate for the epistemologies of the communities involved. Applying a decolonial framework has significant implications for mental health research in Zimbabwe. It challenges the dominance of Western paradigms that often marginalise indigenous knowledge systems. By acknowledging *Ngozi*, the study contributes to unsettling entrenched colonial narratives that disregard the cultural context of mental health. This approach aligns with the call for transformative practices that honour the lived experiences and traditions of non-Western societies. Methodologically, the use of autoethnographic approach in this study has been particularly liberating compared to other methodologies I have used before. Autoethnography allowed me to reflect deeply and incorporate my own voice alongside those of the participants, fostering a richer, more nuanced understanding of *Ngozi* and mental health. This method transcends the limitations of conventional research approaches that often distance the researcher from the subject matter. By embedding myself within the cultural and social fabric of the societies, I could thoroughly engage with the narratives and the subject of the influence of *Ngozi* on mental health.

Considering these reflections and the proposed *Dandemutande* framework, it becomes evident that addressing mental health challenges in Zimbabwe necessitates a paradigm shift, one that honours indigenous knowledge systems while embracing effective contemporary interventions. It is important to understand cultural beliefs, respect those beliefs, and use those beliefs as appropriate to engage someone in treatment. By integrating *Ngozi* into mental health practices, we can foster a more culturally acceptable approach. This integration not only empowers individuals and communities but also challenges colonial legacies that have long marginalised indigenous perspectives.

*Dandemutande* framework represents a promising approach in mental health interventions, offering a model that is both culturally respectful and clinically effective. By embracing indigenous beliefs alongside Western practices, it paves the way for holistic mental healthcare in Zimbabwe.

## Conclusion

Grounded in decoloniality theory (Tuck & Yang 2012), this study challenges Western-centric mental health models, advocating for the validation of indigenous epistemologies. By acknowledging and integrating *Ngozi*, the article contributes to dismantling colonial narratives that overlook the cultural context of mental health. This study illustrates the complex relationship between non-Western beliefs, culture, and mental health in Zimbabwe. Through a multilayered methodology, I explored narratives that highlight the significance of *Ngozi* in relation to mental health challenges. The stories of the man at Chapoto Business centre, the tormented family haunted by the herdsman’s *Ngozi*, and the Chokuda case exemplify how spirituality, collective responsibility, and social justice are deeply intertwined with mental health.

Firstly, *Ngozi* functions as a cultural mechanism enforcing communal accountability, aligning with *Ubuntu* or *Unhu’s* emphasis on interconnectedness and collective well-being. These narratives demonstrate how spiritual beliefs promote social harmony by discouraging actions that disrupt community integrity. Secondly, the spiritual interpretations of mental health highlight the need for culturally sensitive approaches that integrate non-Western understandings with Western or modern mental health practices. Acknowledging that mental distress is also viewed through a spiritual lens allows for interventions that are both respectful and effective within these cultural contexts. Thirdly, the intersection of justice, reconciliation, and mental health highlights the necessity of aligning health and legal frameworks with traditional rituals. The Chokuda case, for example, shows how integrating legal accountability with spiritual reconciliation fosters comprehensive healing and reinforces communal bonds.

The proposed *Dandemutande* [Spiderweb] framework is a transformative model that positions the individual at its centre and weaving together diverse interventions: spiritual, medical, familial, and alternative therapies. This culturally sensitive approach respects and integrates both non-Western and Western practices, recognising that mental health is multifaceted and dynamic. By incorporating the *Ubuntu* or *Unhu* philosophy, the framework emphasises the communal nature of healing and the importance of social support networks, highlighting collective responsibility in promoting mental well-being.

Addressing mental health challenges in Zimbabwe and similar other contexts, requires a paradigm shift that values indigenous knowledge systems while embracing contemporary interventions. The *Dandemutande* framework offers a robust, culturally sensitive model that empowers individuals and communities, fosters holistic healing, and challenges colonial legacies. National leaders must champion this transformation, ensuring that culturally valued practices receive the recognition and support they deserve. Through such commitment, mental health services can become more effective, and respectful of the cultural identities that shape our collective humanity.

Implementing the *Dandemutande* framework has potential significant implications for mental health practice and policy. Policymakers, mental health professionals, and communities must collaborate to decolonise existing laws, incorporate traditional practices, and design culturally compatible interventions. Empowering communities through inclusive policies and education enhances the sustainability and efficacy of mental health initiatives.

## Footnotes

1 The following Shona-language video clips, some of which were presented before local traditional chiefs, highlight cases where *Ngozi* is believed to have manifested: (1) Snodia case (Karanganda TV 2025). A father killed her daughter with his goblins. The daughter’s *Ngozi* then came through her brother’s daughter. She narrated how she was killed. When the case was brought before Chief Saurombe, the father admitted and the *Ngozi* demanded five cows; (2) Tonderai’s case (Karanganda TV 2024). Tonderai was reportedly assaulted by his stepmother after he allegedly damaged a television set while his father was away. The incident ultimately drove him to take his own life. Some time after his death, Tonderai’s *Ngozi* manifested through his brother’s wife, recounting the events leading to his death and demanding rituals and compensation from his stepmother. The case was brought before Chief Mutasa, where the possessed wife narrated and reiterated the spirit’s demands. The stepmother admitted to the allegations.
